# Pancreatic Alpha Cells Hold the Key to Survival

**DOI:** 10.1016/j.ebiom.2015.04.014

**Published:** 2015-04-22

**Authors:** Jibran A. Wali, Helen E. Thomas

**Affiliations:** St Vincent's Institute, Fitzroy, Victoria, 3065, Australia; The University of Melbourne, Department of Medicine, St Vincent's Hospital, Fitzroy, Victoria, 3065, Australia

One of the mysteries in understanding the pathogenesis of diabetes is how glucagon-producing alpha cells in the pancreas remain relatively protected from the toxic environment created by metabolic stress, while insulin-producing beta cells are not protected and die by apoptosis. Metabolic stress refers to conditions of hyperglycemia, or chronically high levels of plasma glucose, and hyperlipidemia that includes increased levels of saturated fatty acids of which palmitate is the most abundant in human plasma. It is believed to be an important cause of beta cell apoptosis in type 2 diabetes. Marroqui and co-workers have made inroads into discovering how alpha cells survive metabolic stress by showing they express higher amounts of survival factors than beta cells (Marroqui et al., 2015).

In vitro, high concentrations of palmitate, glucose, or both combined cause beta cell endoplasmic reticulum (ER) stress and apoptosis. Beta cell ER stress is a known feature of type 2 diabetes in humans and animal models (Laybutt et al., 2007). Cells respond to ER stress by activating the unfolded protein response (UPR) signaling pathway. If the ER stress is mild, UPR signaling results in reduced overall cellular protein synthesis and increased transcription of ER chaperones to relieve the ER stress. However, in severe or prolonged ER stress conditions, pro-apoptotic factors, including CHOP, become activated resulting in cell death.

The evidence for a role for beta cell ER stress in type 2 diabetes comes from electron microscopy of ER appearance, as well as protein and mRNA expression studies in pancreas sections and islets of organ donors with type 2 diabetes (Butler et al., 2003, Laybutt et al., 2007). Using electron microscopy, Marroqui and co-workers observed an increase in the volume density of the ER in both alpha and beta cells in pancreases from type 2 diabetic subjects, a hallmark of the UPR (Marroqui et al., 2015). The authors also used electron microscopy to confirm previous reports using TUNEL staining to show an increase in the number of apoptotic beta cells, but not alpha cells, in pancreases of type 2 diabetic organ donors compared with those from non-diabetic donors (Butler et al., 2003, Rahier et al., 2008). This suggested that while both cell types respond to the metabolic stress conditions of type 2 diabetes, only the beta cells succumb to apoptotic ER stress.

Inulin granules were used as a marker of beta cells in the electron microscopic analysis. Others have suggested that sick beta cells de-differentiate and degranulate in type 2 diabetes and this could be a major mechanism of beta cell failure. The authors of the current study did not comment on whether they saw any apoptotic insulin-negative beta cells. This is an important question because it is not clear if the sick beta cells prolong their survival by de-differentiation, or if this is a step preceding apoptosis.

The authors then went on to study the mechanism of protection of alpha cells from ER stress-induced apoptosis. The Bcl-2-regulated pathway of apoptosis is activated by cellular stresses. It is controlled by the balance between pro-survival and pro-apoptotic members of the Bcl-2 family. The pro-survival proteins include Bcl-2, Bcl-xL, Bcl-w, Mcl-1 and A1. Cellular stress down-regulates these survival factors, allowing translocation of the multi-domain proteins BAX and BAK to the outer mitochondrial membrane where they facilitate apoptosis. The pro-apoptotic BH3-only proteins inhibit the pro-survival members of the family and are required for initiation of apoptosis signaling. Islets isolated from organ donors with type 2 diabetes had elevated mRNA expression of the pro-apoptotic BH3-only molecules BIM (*BCL2L11*) and PUMA (*BBC3*) (Wali et al., 2014), highlighting that their activation is cell type and stimulus-specific.

Marroqui and co-workers observed higher amounts of Bcl-xL (*Bcl2l1*) in alpha cells than in beta cells (Marroqui et al., 2015). Silencing of Bcl-xL with siRNA made alpha cells susceptible to palmitate-induced apoptosis, to the degree observed in beta cells. Silencing another pro-survival protein, Bcl-2, did not alter the sensitivity of alpha cells to palmitate-induced apoptosis. It would be interesting to know whether Mcl-1, another pro-survival protein that is expressed at high levels in islets, also plays a role in alpha cell survival.

The authors conclude that alpha cells are equipped to adapt to and survive metabolic stress by expressing higher expression of survival factors. So why can't beta cells similarly adapt to this stress? Preservation of beta cell mass in diabetes is something all beta cell biologists would like to achieve. The mechanisms of loss of beta cell mass is different in type 1 and type 2 diabetes, but the metabolic outcome is the same, and beta cell ER stress is a feature of both diseases. Could we make beta cells more like alpha cells so they are better able to withstand ER stress-induced apoptosis? Overexpression of pro-survival Bcl-2 family proteins in beta cells using the insulin promoter in transgenic mice (e.g. RIP-Bcl2 or RIP-Bcl-xL) protects them from apoptosis induced by high glucose concentrations or ER stress in vitro (McKenzie et al., 2010, Zhou et al., 2000). However, in vivo, these factors impaired insulin secretion and glucose tolerance, indicating that their overexpression may not be useful for beta cells in conditions of metabolic stress (Luciani et al., 2013, Zhou et al., 2000).

Alpha cells are also preserved in type 1 diabetes. This is at least in part due to the specificity of the immune response to beta cell antigens such as proinsulin. However, the islets are infiltrated by immune cells that secrete a range of cytotoxic proteins that would presumably affect alpha cells in a bystander manner. The current study therefore raises the possibility that alpha cells may also survive better in an inflammatory environment such as in type 1 diabetes. Indeed in a recent proteomic study of alpha and beta cell lines, a major difference was observed in the defense of alpha cells and beta cells against reactive oxygen species due to differences in expression of superoxide dismutase 2, a major scavenging enzyme (Gorasia et al., 2015).

It is therefore likely that alpha cells are equipped with multiple mechanisms that maintain their survival in diabetic conditions. Alpha cells have recently been touted as having a guardian-type role in the islet to maintain the body's capacity to produce insulin (Habener and Stanojevic, 2013). There is an emerging role for alpha cells in transdifferentiation into beta cells, in addition to production of GLP-1, a factor that stimulates insulin secretion as well as beta cell survival. It therefore makes sense for these cells to be able to survive metabolic stress so they can restore insulin secretory function if necessary. It would be interesting to see whether alpha cells that have transdifferentiated into beta cells maintain their improved capacity for survival.

## Conflict of interest statement

The authors declare no conflict of interest.
